# The effect of client-auditor mismatches on earnings management using classification shifting: Evidence from China

**DOI:** 10.1371/journal.pone.0344850

**Published:** 2026-03-20

**Authors:** Shanmei Luo, Danning Yu, Shudan Jin

**Affiliations:** Business School, Shaoxing University, Shaoxing City, Zhejiang Province, China; Liverpool John Moores University, UNITED KINGDOM OF GREAT BRITAIN AND NORTHERN IRELAND

## Abstract

Using a sample of Shanghai and Shenzhen A-share listed companies from 2008 to 2022, this study examines the impact of client-auditor mismatch on earnings management using classification shifting. The results indicate that upward mismatches—where clients engage higher-tier auditors than expected—mitigate classification shifting, while downward mismatches amplify it. Further analysis reveals that strong internal control moderates this relationship, constraining earnings management behavior. Additionally, we find that auditor industry expertise partially mediates the relationship between client-auditor mismatch and classification shifting.

## 1. Introduction

Independent auditing plays a critical role as the “economic police” within the capital market supervision system, serving as an external mechanism for evaluating the credibility of disclosed financial reports and safeguarding the orderly functioning of capital markets. Auditors act as independent appraisers of financial information, and thus constituting an essential pillar of market-based governance. However, the frequent exposure of high-profile financial fraud cases, such as Kangde Xin and Kangmei Pharmaceutical, has raised serious concerns among investors regarding the effectiveness of audit supervision, prompting both regulators and academics to re-examine the nature of the client–auditor relationship.The employment dynamic between these two parties reflects the supply-demand relationship in the auditing market, directly influencing the quality of audit supervision. Therefore, maintaining a sound client-auditor relationship, safeguarding audit independence, and delivering high-quality audit services are of great practical significance for promoting the healthy development of the audit market.

High-quality financial reporting is a fundamental requirement for the healthy development of capital markets. At its core, high-quality reporting aims to reduce information asymmetry through the disclosure of truthful and fair financial information, thereby providing a sound basis for resource allocation decisions [1]. However, the widespread presence of earnings management directly undermines the reliability and decision-usefulness of financial information, posing a significant obstacle to the efficient functioning of capital markets.

There are three types of earnings management: accrual-based earnings management, real activity earnings management, and earnings management using classification shifting. Among them, earnings management using classification shifting is a relatively special form of earnings management characterized by low cost and strong concealment, through which management can pursue personal gains by manipulating core profits [2]. Unlike traditional strategies that increase or decrease total earnings, classification shifting operates by altering the structure of earnings rather than their magnitude. Specifically, firms may misclassify non-core income (e.g., gains on asset disposals or government subsidies) as core operating income, or shift recurring expenses (e.g., selling and marketing expenses) to the non-recurring category. Such practices mislead investors’ perceptions of a firm’s “sustainable profitability” [3].

In China, classification shifting is prevalent among listed companies. According to available statistics, from 2019 to 2023, 41% of firms subject to delisting risk warnings (“ST” status) reported non-recurring items accounting for more than 50% of core earnings in the year prior to being removed from ST status—a figure that far exceeds the 12% observed in non-ST firms. Furthermore, between 2022 and 2023, the Shanghai and Shenzhen Stock Exchanges issued over 2,300 regulatory inquiries regarding classification issues such as the presentation of non-recurring gains and the capitalization of R&D expenditures. These inquiries accounted for 39% of all financial–related inquiries issued during the same period. The above data are obtained from the official websites of the Shanghai and Shenzhen Stock Exchanges.

The concealment of classification shifting makes it an effective “grey-area tool” for managers to avoid regulatory scrutiny and maintain stock price stability. Core earnings serve as a key indicator used by investors to assess firms’ long-term performance potential [4]; therefore, their reliability has direct implications for capital pricing efficiency. Dechow et al. [5] find that classification-shifted core earnings significantly overstate future profitability, thereby leading to stock mispricing. When the temporary “window-dressing” effects of classification shifting fade over time—such as when non-core gains prove unsustainable—investors often suffer excess losses due to unmet expectations, which in turn exacerbates market volatility [6]. Consequently, identifying and addressing classification shifting has become a pressing issue not only in financial reporting governance, but also in the broader context of capital market efficiency.

Beyond its implications for audit quality and corporate governance, earnings management—particularly through classification shifting—also shapes how financial information is interpreted by investors. Although classification shifting does not alter net income, core earnings play a critical role in shaping market perceptions of a firm’s sustainable performance. Prior research indicates that such earnings manipulations impair the value relevance of financial statements, misleading investors and distorting stock valuations. For instance, Rawashdeh et al. [7] and Burlacu et al. [8] document that earnings management weakens the association between earnings and stock prices, while Garel [9] further highlights that short-term market pressures may induce managerial myopia, encouraging the use of cosmetic reporting tactics to temporarily inflate firm value. Taken together, these findings suggest that the consequences of classification shifting extend beyond financial reporting quality to affect market pricing and investor welfare. Accordingly, this study not only contributes to the literature on auditors and earnings management, but also offers new insights into the capital market implications of subtle yet impactful earnings manipulation strategies such as classification shifting.

Although a large number of literatures have studied the auditors and earnings management respectively [10,11], few literatures have studied the influence and interaction mechanism between Client-Auditor Mismatches and classification shifting. In order to fill this gap, this paper examines the impact of client-auditor mismatch on earnings management using classification shifting.

Client-auditor mismatches can be classified into downward and upward mismatches. Downward mismatch occurs when the auditor selected by the client lacks sufficient resources, expertise, or service capacity to cover the client’s business complexity and risk profile. This situation often arises when large-scale or complex firms engage audit firms with limited professional teams or experience in handling complex transactions. In such cases, auditors’ inability to fully identify potential risks increases information asymmetry and reduces the likelihood of detecting subtle earnings manipulation, creating incentives for managers to engage in classification-shifting earnings management.

Upward mismatch occurs when the auditor selected exceeds the client’s actual needs, meaning the client’s resources, business complexity, or risk exposure are lower than the auditor’s service standards and resource requirements. This is commonly observed when small- or medium-sized firms engage Big Four or top domestic auditors. They choose high-quality auditors for their own reputation (Fan and Wong, 2005) [12]. In such cases, auditors can discover earnings management behavior by adopting standardized procedures, thus reducing the motivation of classified transfer to manipulate income.

The economic and organizational rationale for these effects can be understood through agency theory and audit quality frameworks. Under downward mismatches, the combination of higher information asymmetry and weaker monitoring fosters managerial opportunism, whereas upward mismatches enhance auditor supervision and constrain earnings management. These mechanisms highlight how the interaction between auditor capabilities, client characteristics, and the regulatory environment shapes managerial behavior, particularly in the Chinese institutional context.

The contributions of this study are summarized in the following aspects: First, from the perspective of the client-auditor supply and demand relationship, the study examines its effect on earnings management using classification shifting, expands the literature on the economic consequences of the relationship between clients and auditors, and offers a new understanding to the selection of auditors by listed companies. Second, while prior studies have mostly focused on the external governance effects of companies from the perspective of audit supply and demand, and they have largely overlooked earnings management issues arising from the bilateral selection game between firms and audit providers. This study has important practical value for improving corporate external governance. Third, this study clarifies both the effect and the underlying mechanism through which client–auditor relationships influence earnings management via classification shifting. This clarification offers a new perspective for the governance of earnings management and provides valuable reference implications for the healthy development of China’s securities market.

## 2. Literature review

### 2.1. The matching relationship between clients and auditors

The matching relationship between clients and auditors is reflected in the degree of alignment, which refers to how well a company is matched with the accounting firm it hires. A successful match between a client and an auditor occurs when, given the constraints of the auditor’s conditions, the services provided by the auditor precisely meet the client’s needs [13]. Different companies have different needs, and different firms have varying abilities to meet these needs, leading to differences in the degree of client-auditor match [13,14]. As the audit market continues to improve and industry competitors emerge, clients’ audit preferences also change, and firms need to adjust their service levels to adapt to industry development, resulting in a continuous adjustment process between supply and demand [15]. These adjustments, whether caused by changes on the demand side or the supply side, can lead to mismatches between the two parties. This phenomenon is known as the client-auditor mismatch [13,16].

Current research on the client-auditor relationship mainly focuses on the social relationship between the auditor and the client, the geographic distance between them, and their matching relationship. Bruynseels [17] found that when there is a social network relationship between the external auditor and the company’s audit committee, it can impair independence and reduce audit quality. Similarly, private relationships between auditors and client alumni yield similar results [18]. Furthermore, the closer the geographic location between the client and the auditor, the more it facilitates the auditor’s understanding of the client, thereby enhancing audit quality [19,20]. A few scholars have studied the matching relationship between clients and auditors. Brown and Knechel [13] found that a compatible match between the client and the auditor can reduce accrual-based earnings management and make it less likely to receive a going concern audit opinion. However, when a mismatch occurs between the auditor and the client, it can harm audit quality, increase the risk of stock price crashes, and lead to more goodwill impairments [21–23].

### 2.2. Earnings management and value relevance of financial information

A growing body of literature has investigated how earnings management affects the value relevance of accounting information. Value relevance refers to the extent to which financial statement information is associated with a firm’s market value. When managers manipulate earnings, especially through covert means such as classification shifting, the informational reliability of financial reports is compromised.

Rawashdeh et al. [7] provide empirical evidence from Jordan showing that earnings management practices weaken the relationship between reported earnings and stock prices, undermining investor confidence in financial disclosures. Similarly, Burlacu et al. [8] demonstrate that earnings management negatively influences the value relevance of financial information among firms listed on the Bucharest Stock Exchange. Liu and Sun [24], focusing on U.S. firms during the COVID-19 pandemic, find that income-decreasing earnings management lowers the slope and significance of earnings coefficients in valuation regressions, further supporting the negative link between earnings management and value relevance.

Meanwhile, Garel [9] explores the behavioral foundations of earnings management and finds that short-term-oriented investors and performance-based compensation schemes can prompt managerial myopia, resulting in earnings management practices aimed at meeting market expectations. Ha and Thomas [25] extend this line of research by specifically examining classification shifting, concluding that it reduces earnings predictability and the reliability of reported operating performance.

### 2.3. Earnings management using classification shifting

Research on earnings management has primarily focused on accrual-based and real activities earnings management, with relatively little attention given to earnings management using classification shifting. Earnings management using classification shifting occurs when a company incorrectly categorizes recurring expenses, or misclassifies non-recurring losses or non-recurring gains as recurring income. This manipulation is highly covert, altering only the company’s core earnings without affecting its net profit [3]. Since core earnings can reflect the true state of a company’s development and influence stock price fluctuations, they have become an important tool for management in earnings manipulation [26]. Rehman et al. [27] suggest that classification earnings management exists among listed companies in China, motivated by the desire to increase operating profit, and they found that corporate governance mechanisms can weaken this phenomenon High-quality auditors, board governance, analyst coverage, and the adoption of international financial reporting standards, as external governance mechanisms, can help mitigate classification shifting earnings management behavior in listed companies [27,28].

Overall, these studies suggest that earnings management poses a significant threat not only to internal governance and audit quality but also to capital market efficiency by impairing the usefulness of reported earnings. Reviewing the above research, it is evident that a small number of studies have examined the impact of the supply-demand relationship between clients and auditors on stock price crash risk, audit quality, and goodwill impairment, but related research remains limited. Few studies have explored the impact of the client-auditor supply-demand relationship on corporate earnings management using classification shifting, especially as core earnings are becoming increasingly important, and earnings management using classification shifting is receiving more attention from various sectors. Based on this, this paper investigates the impact of the client-auditor mismatch on corporate earnings management using classification shifting and analyzes its influence pathways, aiming to provide new insights for the governance of earnings management.

## 3. Motivation and hypothesis development

According to agency theory, significant agency conflicts exist between company managers and shareholders because their interests are not fully aligned. To meet performance evaluation targets, managers may engage in earnings management to manipulate reported earnings and adjust perceived firm performance. Earnings management using classification shifting is a specific type of earnings management that involves misclassifying routine operating expenses and non-operating income by shifting them between different line items on the income statement. This type of vertical reclassification does not alter total earnings and is generally not expected to have a direct adverse impact on a firm’s future operations or long-term development. Compared to accrual-based earnings management and real activities earnings management, classification shifting is relatively easier to implement, more difficult to detect, and therefore more likely to be favored by management [28]. As the external regulatory environment becomes stricter and accounting standards continue to improve, the opportunities for accrual-based earnings management have been greatly reduced, making it increasingly difficult to execute. Real activities earnings management, which requires adjustments to actual business operations, can damage company value and has significant negative implications for the long-term development of the enterprise. Consequently, firms are more inclined to rely on the more covert and less disruptive practice of classification shifting to achieve earnings targets [27].

As a key component of external corporate governance, auditors, being independent of the company, can effectively identify client earnings management behaviors by reviewing relevant data and materials in the financial statements [29]. Audit quality varies across audit firms. Large audit firms generally possess greater industry expertise, more extensive industry experience, and specialized audit resources and information technologies tailored to specific industries, which collectively enhance their independence and professionalism [30]. When large audit firms provide audit services to relatively small firms, an upward client–auditor mismatch arises. In this situation, for large auditors, the smaller business volume and lower complexity of small companies mean that auditors have ample time to understand the client and implement various audit procedures, such as analytical procedures and control tests, making them more likely to identify misclassifications among accounting items and thereby reduce earnings management using classification shifting.

Industry expertise represents a key dimension of auditors’ professional competence, and large firms typically possess higher industry expertise, meaning that large auditors have greater professional competence [31]. Large auditors use their industry expertise to understand the client’s characteristics and operating environment, and compare and review the routine operating expenses with non-operating income, making it easier to detect management’s classification-shifting behaviors. Additionally, large firms, in order to maintain a good reputation, tend to be more cautious during the audit process, adopt a low tolerance for opportunistic behaviors by management, and conduct strict reviews of financial reports, which can reduce management’s earnings management using classification shifting. Based on the above analysis, the following hypothesis is proposed.

***H1.*** An upward mismatch between the client and the auditor is negatively associated with a firm’s earnings management using classification shifting.

When there is a downward mismatch between the client and the auditor, it means that a small audit firm has taken on the business of a large-scale enterprise. From the perspective of professional competence, when small firms take on audit engagements beyond their capacity, they may be unable to effectively perform audits for large firms, making it difficult to fully fulfill auditing and supervisory functions. Small firms have limited audit scale and resources, and when they take on large, complex business engagements, they may struggle to quickly and accurately assess the client’s financial condition and potential operating risks, leading to compromised audit quality [30]. Since earnings management using classification shifting does not change the company’s net profit or actual operating conditions, it is difficult to detect. When small auditors take on large engagements, the audit risk is higher, and they must deal with more complex activities. Due to limited professional competence, they are less likely to detect misclassifications among accounting items, making it difficult to exercise their supervisory role and possibly leading to an increase in management’s classification-shifting activities.

From the perspective of audit independence, compared to large auditors, small auditors face lower potential costs or losses from impairments to independence.. Therefore, small auditors are more likely to sacrifice independence for the economic benefits brought by large clients [32]. In cases where there is a downward mismatch between the client and the auditor, small auditors become more economically dependent on large clients in order to retain them [32]. In the current competitive audit market environment, strong economic dependence may lead auditors to compromise with large clients, thereby damaging audit quality [33,34]. Therefore, when small auditors take on large-scale audit engagements, they may relax materiality thresholds to preserve client relationships, especially with economically important clients. which reduces their incentives to disclose or challenge the client’s earnings management using classification-shifting practices. Based on the above analysis, the following hypothesis is proposed.

***H2.*** A downward mismatch between the client and the auditor is positively associated with a firm’s earnings management using classification shifting practices.

## 4. Research design

### 4.1. Sample selection and data sources

The sample selected for this study consists of data from A-share listed companies on the Shanghai and Shenzhen stock exchanges in China from 2008 to 2022. Following the methodologies of related studies, the sample was screened and processed, resulting in a final dataset of 32,416 observations. All relevant data were downloaded from the CSMAR database and CICPA (The Chinese Institute of Certified Public Accountants) website. All continuous variables in the regression process were treated with trimming and Winsorizing.

### 4.2. Variable design and model design

#### 4.2.1. Key variable definitions.

(1) Explanatory Variable: Client-Auditor Mismatch Variable

Building on the work of Shu [35] and Lai and Leung [16], Model (1) was developed to assess the client-auditor mismatch relationship.


$$Big10_{i,t}=\partial_{\text{0}}+\partial_{\text{1}}Size_{i,t}+\partial_{\text{2}}Lev_{i,t}+\partial_{\text{3}}Atu_{i,t}+\partial_{\text{4}}Cr_{i,t}+\partial_{\text{5}}Roa_{i,t}+\Sigma Ind_{i,t}+\Sigma Year_{i,t}+\varepsilon_{i,t}$$ (1)

In model (1), *Big10* is measured based on the ranking of the top 100 firms on the CICPA website, where a value of 1 is assigned if the client hires one of the top 10 firms, and 0 otherwise. The definitions of other specific variables are provided in the table of key variable definitions.

This study estimates model (1) and uses the fitted values obtained from the equation as the probability of a client hiring one of the Big 10 accounting firms. Additionally, the optimal threshold probability of 0.484 was calculated across the sample companies over the years. If a company’s probability of hiring one of the Big 10 domestic accounting firms exceeds this threshold, the company is classified as a potential client of the Big 10 accounting firms. Conversely, if the probability is below the threshold, the company is classified as a non-potential client of the Big 10 accounting firms. The ratio of a company’s actual selection value to its expected selection value of an accounting firm is used as the measure of the client-auditor mismatch relationship. If a potential client actually chooses a non-Big 10 accounting firm, it is classified as a downward mismatch; conversely, if a non-potential client actually chooses a Big 10 accounting firm, it is classified as an upward mismatch. We construct two indicator variables to capture these two types of mismatches. *Misup* is equal to 1 if a firm with Prb10 ≤ Critical Probability (i.e., not expected to choose a Big 10 accounting firm) actually selects one, and 0 otherwise. *Misdown* is equal to 1 if a firm with Prb10 > Critical Probability (i.e., expected to choose a Big 10 accounting firm) instead selects a non-Big 10 firm, and 0 otherwise. When the actual choice aligns with the expected choice, both *Misup* and *Misdown* are set to 0. The construction of these variables is summarized in Table 1.

**Table 1:** Value Assignment for Client-Auditor Mismatch Relationship.

*Big10* (Actual choice)	Prb10 (Expected choice)	*Misup*	*Misdown*
1	≤Critical probability	1	0
0	>Critical probability	0	1
1	>Critical probability	0	0
0	≤Critical probability	0	0

Specifically, the calculation of the cutoff value follows the approach proposed by Shu [35]. The procedure first identifies the type of auditor that a client is potentially expected to choose and then compares this expected choice with the client’s actual auditor selection. A Logit model is employed to estimate the probability that a client selects a large audit firm (Big 10), yielding a predicted probability for each observation. We calculate Type I errors (potential Big 10 clients choosing Non-Big 10 auditors) and Type II errors (potential Non-Big 10 clients choosing Big 10 auditors) at each predicted probability level. The predicted probabilities are then ordered, and the probability value corresponding to the minimum total number of Type I and Type II errors is selected as the cutoff threshold. This variable compares a client’s expected auditor choice with its actual auditor selection, thereby precisely capturing the systematic mismatch arising from supply–demand misalignment in the audit market.

(2) Dependent Variable: Earnings Management Using Classification Shifting

Following the method of McVay [3] and Liu et al. [36], the expected core earnings are first predicted. Then, residual values are regressed by industry and by year to estimate the level of unexpected core earnings (*UE_CE*). Specifically, Equation (2) is estimated separately for each year and industry, and the residuals from these regressions are taken as the measure of unexpected core earnings (*UE_CE*).


$$Ce_{t}=\beta_{\text{0}}+\beta_{\text{1}}Ce_{t-1}+\beta_{\text{2}}Ato_{t}+\beta_{\text{3}}Accruals_{t-1}+\beta_{\text{4}}Accruals_{t}+\beta_{\text{5}}\Delta Sales_{t}+\beta_{\text{6}}Neg\_\Delta Sales_{t}+\varepsilon_{\text{1}}$$ (2)

In Equation (2), the variable *Ce* represents core earnings standardized by the beginning total assets of the current period. Core earnings are calculated as: (Operating Revenue – Operating Costs – Period Expenses)/ Operating Revenue. The variable *Atot* denotes the total asset turnover ratio. *Accruals* captures accrual-based earnings for the current period. *ΔSales* is measured as the growth rate of sales revenue. The variable *Neg_*Δ*Sales* is a dummy variable indicating a decline in sales revenue. It takes the value of 1 if the sales growth rate in the current period is negative, and 0 otherwise.

#### 4.2.2. Control variables.

The control variables in this study include company size, leverage ratio, CEO duality, management ownership, market-to-book ratio, the proportion of shares held by the largest shareholder, ownership concentration, board size, the proportion of independent directors, and audit opinion. The table below provides specific definitions for these variables.

### 4.3. Multiple regression model

This study examines the impact of the client-auditor mismatch on classification shifting in earnings management, based on the relationship between the client and the auditor. To test the hypothesis, the article constructs the multiple regression model (3) as follows:


$$\begin{array}{c} UE\_CE=\partial_{\text{0}}+\partial_{\text{1}}Mi{sup}_{i,t}+\partial_{\text{2}}Misdown_{i,t}+\partial_{\text{3}}Size_{i,t}+\partial_{\text{4}}Lev_{i,t}+\partial_{\text{5}}Dual_{i,t}+\\ \partial_{\text{6}}Mborate_{i,t}+\partial_{\text{7}}Mb_{i,t}+\partial_{\text{8}}Firstshare_{i,t}+\partial_{\text{9}}Shrcr_{i,t}+\partial_{10}Boardsize_{i,t}+\partial_{11}Indpend_{i,t}\\ +\partial_{12}Opinion_{i,t}+\varepsilon_{i,t} \end{array}$$ (3)

where *UE_CE* represents earnings management using classification shifting; $\partial_{\text{0}}$ is the constant term; $\partial_{\text{1}}$ and $\partial_{\text{2}}$ are the regression coefficients of the explanatory variables;  $\partial_{\text{3}}$~ $\partial_{12}$ are the regression coefficients of the control variables; $\varepsilon_{i,t}$ represents the error term. The coefficient $\partial_{\text{1}}$ is expected to be significantly negative, while $\partial_{\text{2}}$ is expected to be significantly positive (Table 2).

**Table 2:** Variables Definition.

Variable Type	Name	Symbol	Definition	Source
Dependent Variable	*Earnings Management Using Classification Shifting*	*UE_CE*	Unexpected Core Earnings Level.	CSMAR
Independent Variables	*Client-Auditor Upward Mismatch*	*Misup*	A value of 1 if the client, who is not a potential client, actually hires one of the Big 10 domestic auditors; otherwise, the value is 0.	CSMAR， CICPA
	*Client-Auditor Downward Mismatch*	*Misdown*	A value of 1 if the potential client actually hires a non-top-ten domestic auditor; otherwise, the value is 0.	CSMAR， CICPA
Control Variables	*Firm Size*	*Size*	The logarithm of the company’s total assets.	CSMAR
	*Leverage Ratio*	*Lev*	The ratio of total liabilities to total assets.	CSMAR
	*Dual Roles in the Company*	*Dual*	A value of 1 if the chairman also serves as CEO; otherwise, the value is 0.	CSMAR
	*Management Shareholding Ratio*	*Mborate*	The ratio of shares held by the company’s management to the total number of shares.	CSMAR
	*Market-to-Book Ratio*	*Mb*	The ratio of the company’s market value to net assets at year-end.	CSMAR
	*Largest Shareholder Ownership Ratio*	*Firstshare*	The ratio of shares held by the largest shareholder to the total number of shares in the company.	CSMAR
	*Ownership Concentration*	*Shrcr*	The sum of the shareholding ratios of the top 10 shareholders.	CSMAR
	*Board Size*	*Boardsize*	The logarithm of the number of board members.	CSMAR
	*Proportion of Independent Directors*	*Indpend*	The ratio of independent directors to the total number of board members.	CSMAR
	*Audit Opinion*	*Opinion*	A value of 0 if the company receives a standard audit opinion for the year; otherwise, the value is 1.	CSMAR, CICPA

## 5. Empirical results

### 5.1. Descriptive statistics

Table 3 presents the descriptive statistics of the main variables. The results show that the dependent variable, *UE_CE*, has a maximum value of 0.451 and a minimum value of −0.777, indicating the presence of earnings management using classification shifting among listed companies, with both upward and downward shifts occurring. The mean value of the client-auditor upward mismatch relationship is 0.158, while the mean value of the downward mismatch relationship is 0.262, suggesting that 15.9% of the companies experience upward mismatch, and 26.2% experience downward mismatch.

**Table 3:** Descriptive Statistics.

Variable	N	Mean	SD	Min	Max
*UE_CE*	32416	0.030	0.159	−0.777	0.451
*Misup*	32416	0.158	0.366	0	1
*Misdown*	32416	0.262	0.440	0	1
*Size*	32416	21.990	1.289	19.383	25.988
*Lev*	32416	0.436	0.210	0.061	0.933
*Dual*	32416	0.270	0.444	0	1
*Mborate*	32416	12.456	19.169	0	70.700
*Mb*	32416	4.190	3.518	1.122	27.429
*Firstshare*	32416	34.147	14.844	8.730	75.170
*Shrcr*	32416	57.654	15.321	22.760	95.360
*Boardsize*	32416	2.131	0.199	1.609	2.708
*Indpend*	32416	37.444	5.316	30.770	57.140
*Opinion*	32416	0.038	0.191	0	1

As for the control variables, the company size (*Size*) ranges from a maximum of 25.988 to a minimum of 19.383, indicating a significant variation in the scale of the companies in the sample. The management ownership ratio (*Mborate*) has a maximum of 70.700% and a minimum of 0, showing substantial differences in management ownership, with some companies even having no management ownership. The descriptive statistics for other control variables also fall within the normal range.

**Fig 1:**
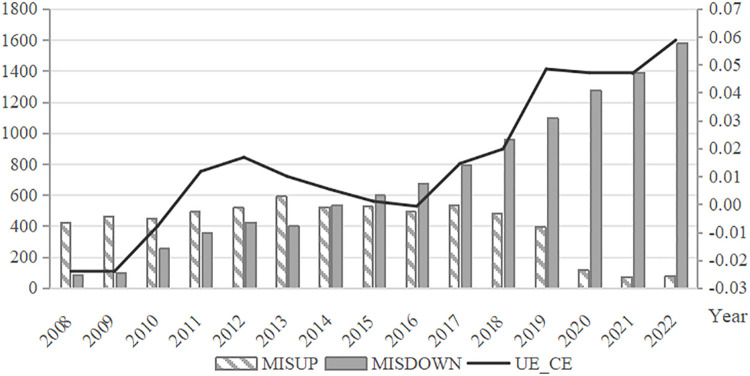
Annual Distribution of *MISDOWN*, *MISUP,* and *UE_CE.*This figure illustrates the annual trends in the number of downward mismatches (*MISDOWN*) and upward mismatches (*MISUP*), as well as the mean value of unexpected core earnings (*UE_CE*), among Chinese A-share listed firms from 2008 to 2022. The number of *MISUP* firms shows a steady decline over time, whereas *MISDOWN* firms exhibit a rising trend, indicating an increasing prevalence of downward auditor-client mismatches in the market. The line plot also shows a gradual increase in classification-shifting earnings management over the sample period.

**Fig 2:**
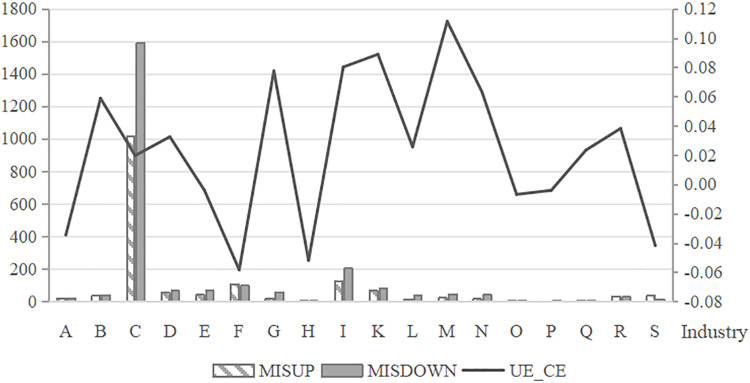
Industry-wise Distribution of *MISDOWN*, *MISUP,* and *UE_CE*. This figure presents the distribution of downward mismatches (*MISDOWN*), upward mismatches (*MISUP*), and mean unexpected core earnings (*UE_CE*) across industries. Downward and upward mismatches are most prevalent in the manufacturing industry, while classification-shifting earnings management is notably concentrated in the scientific research and technical services industry. Note: A: Agriculture, Forestry, Animal Husbandry, and Fishery; B: Mining; C: Manufacturing; D: Production and Supply of Electricity, Heat, Gas, and Water; E: Construction; F: Wholesale and Retail Trade; G: Transportation, Storage, and Postal Services; H: Accommodation and Catering Services; I: Information Transmission, Software, and Information Technology Services; K: Real Estate; L: Leasing and Business Services; M: Scientific Research and Technical Services; N: Water Conservancy, Environment, and Public Facilities Management; O: Resident Services, Repairs, and Other Services; P: Education; Q: Health and Social Work; R: Culture, Sports, and Entertainment; S: Public Administration, Social Security, and Social Organizations.

The study further examines the annual and industry-level trends in the number of *MISDOWN* and *MISUP* firms, as well as the distributional characteristics of the mean *UE_CE* values among listed companies from 2008 to 2022. As shown in Fig 1, which depicts the annual trends, the number of upward mismatch (*MISUP*) firms has been decreasing year by year, while the number of downward mismatch (*MISDOWN*) firms has been on the rise. This suggests a growing prevalence of auditor-client mismatch in the auditor selection practices of Chinese listed companies. The line graph in the figure illustrates the trend of classification shifting earnings management, revealing a gradual increase in such activity over the observed period. Fig 2 presents the trends across industries. It shows that both upward and downward mismatches are more prevalent in the manufacturing industry, while classification shifting earnings management is more common in the scientific research and technical services industry.

The study divides the sample into two groups based on the type of client-auditor mismatch and performs *t*-tests for mean differences and z-tests for median differences of relevant variables within each group. The data in Table 4 reveals that when there is an upward mismatch between the client and the auditor, the level of earnings management using classification shifting is significantly lower than in other companies. Conversely, when there is a downward mismatch, the level of earnings management using classification shifting is significantly higher. Additionally, the *t*-values and *z*-values for the mean and median difference tests between the two groups are significant, indicating a clear distinction between the matched and mismatched samples.

**Table 4:** Testing Differences Between Matching and Non-Matching Groups.

	Mismatch = 1		Mismatch = 0		*t-values the mean difference tests*	*t-values for the mean difference tests*
	Mean	Med.	Mean	Med.		
*UE_CE*	0.026	0.019	0.032	0.026	0.005***	23.616***
*Misu*	0.053	0.000	0.119	0.000	−0.066***	495.439***
*Misd*	0.105	0.000	0.615	1.000	−0.509***	134.350***
*Size*	21.922	21.792	22.040	21.837	0.118***	7.909***
*Lev*	0.432	0.425	0.439	0.432	0.007***	4.009**
*Dual*	0.267	0.000	0.273	0.000	0.006	1.216
*Mborate*	12.437	0.306	12.469	0.252	0.032	2.205
*Mb*	4.172	3.262	4.203	3.226	0.031	2.552
*Firstshare*	33.904	31.450	34.324	32.105	0.420***	7.645***
*Shrcr*	57.140	57.800	58.028	58.880	0.888***	19.030***
*Boardsize*	2.132	2.197	2.131	2.197	−0.002	0.465
*Indpend*	37.382	33.330	37.489	35.710	0.107*	1.650
*Opinion*	0.037	0.000	0.039	0.000	0.002	0.649

***, **, * Indicate significance levels at 0.01, 0.05, and 0.10, respectively. See Table 2 for variable definitions.

### 5.2. Correlation analysis

Before conducting regression analysis, this study performed a correlation analysis of the variables. As shown in Table 5, there is a potential negative correlation between *UE_CE* and the client-auditor upward mismatch relationship (*Misup*), and a potential positive correlation between UE_CE and the client-auditor downward mismatch relationship (*Misdown*). Both correlations are significant at the 1% level. This suggests that companies choosing high-quality audit firms tend to suppress earnings management using classification shifting, whereas selecting lower-quality audit firms tends to exacerbate this behavior.

**Table 5:** Correlation Matrix.

	*UE_CE*	*Misup*	*Misdown*	*Size*	*Lev*	*Dual*	*Mborate*	*Mb*	*Firstshare*	*Shrcr*	*Boardsize*	*Indpend*	*Opinion*
*UE_CE*	1												
*Misup*	−0.109***	1											
*Misdown*	0.082***	−0.192***	1										
*Size*	0.040***	−0.241***	0.150***	1									
*Lev*	−0.355***	0.041***	−0.061***	0.401***	1								
*Dual*	0.069***	0.007	−0.010**	−0.161***	−0.159***	1							
*Mborate*	0.200***	−0.006	0.016***	−0.288***	−0.344***	0.267***	1						
*Mb*	−0.193***	0.097***	−0.086***	−0.130***	0.382***	−0.020***	−0.159***	1					
*Firstshare*	0.099***	−0.035**	0.013***	0.190***	0.026***	−0.050***	−0.056***	−0.093***	1				
*Shrcr*	0.227***	−0.075***	0.028	0.153***	−0.146***	0.061***	0.271***	−0.185***	0.621***	1			
*Boardsize*	−0.003	−0.013***	0.011**	0.251***	0.146***	−0.184***	−0.200***	−0.029***	0.020***	0.016***	1		
*Indpend*	0.006	−0.009**	−0.006	0.014***	−0.012**	0.111***	0.077***	0.024**	0.035***	0.027***	−0.518***	1	
*Opinion*	−0.307***	0.054***	−0.050***	−0.103***	0.211***	−0.007	−0.089***	0.219***	−0.112***	−0.140***	−0.040***	0.017***	1

***, **, * Indicate significance levels at 0.01, 0.05, and 0.10, respectively. See Table 2 for variable definitions.

Given the numerous factors that influence earnings management using classification shifting, further regression analysis is necessary. The study also calculated the variance inflation factor (VIF) values for the variables, with all VIF values being below 2 and an average VIF of 1.47. This indicates that there are no serious multicollinearity issues among the variables.

### 5.3. Multiple regression analysis

This study conducted multiple regression analyses on Model (3) to examine the impact of the client-auditor mismatch relationship on earnings management using classification shifting. As shown in Table 6, the coefficient for *Misup* is −0.017, which is significantly negatively correlated with earnings management using classification shifting at the 1% level. This indicates that an upward mismatch between the client and auditor suppresses classification shifting, supporting Hypothesis 1. On the other hand, the coefficient for *Misdown* is 0.007, which is significantly positively correlated with classification shifting at the 1% level. This suggests that a downward mismatch between the client and auditor exacerbates classification shifting, supporting Hypothesis 2.

**Table 6:** Client-Auditor Mismatch and Earnings Management Using Classification Shifting.

	(1)	(2)
	*UE_CE*	*UE_CE*
*Misup*	−0.017***	
	(−7.515)	
*Misdown*		0.007***
		(3.977)
*Size*	0.020***	0.021***
	(25.084)	(26.470)
*Lev*	−0.295***	−0.297***
	(−39.560)	(−40.084)
*Dual*	0.010***	0.010***
	(5.627)	(5.557)
*Mborate*	0.001***	0.001***
	(18.028)	(17.982)
*Mb*	0.002***	0.002***
	(8.923)	(8.973)
*Firstshare*	−0.000**	−0.000**
	(−2.152)	(−2.107)
*Shrcr*	0.001***	0.001***
	(18.978)	(18.954)
*Boardsize*	0.014***	0.013***
	(2.880)	(2.815)
*Indpend*	−0.000	−0.000
	(−0.475)	(−0.527)
*Opinion*	−0.159***	−0.159***
	(−39.168)	(−39.154)
*Constant*	−0.441***	−0.462***
	(−21.405)	(−22.711)
*Year Fixed Effects*	Yes	Yes
*Industry Fixed Effects*	Yes	Yes
*N*	32416	32416
*Adj.R2*	0.288	0.288

***, **, * Indicate significance levels at 0.01, 0.05, and 0.10, respectively. See Table 2 for variable definitions.

One possible explanation is that when there is an upward mismatch, large audit firms are matched with smaller enterprises, and these larger auditors, with their higher industry expertise, are more likely to detect classification-shifting activities. Conversely, when there is a downward mismatch, larger enterprises are matched with smaller audit firms. These smaller auditors have limitations in audit quality, experience, and resources, making it difficult for them to identify earnings management using classification shifting.

Among the control variables, company size (*Size*) is significantly positively correlated with classification shifting, indicating that such behavior is more prevalent in larger companies. Leverage ratio (*Lev*) is significantly negatively correlated with classification shifting, suggesting that higher financial leverage significantly restrains the occurrence of classification shifting. The management shareholding ratio (*Mborate*) is significantly positively correlated, indicating that an increase in management’s shareholding ratio intensifies classification shifting. Lastly, audit opinion (*Opinion*) is significantly negatively correlated with classification shifting, implying that issuing non-standard audit opinions helps reduce classification-shifting activities.

To address potential concerns related to serial correlation and heteroskedasticity at the firm level, we re-estimate the baseline regressions by adjusting standard errors for clustering at the firm and industry levels. The clustering-adjusted results are reported in a separate robustness section (Section 6.7). After applying clustered standard errors, the signs, statistical significance, and economic magnitudes of the coefficients on the core explanatory variables remain largely unchanged. These findings indicate that our baseline conclusions are not driven by misspecified standard errors and are robust to alternative inference procedures.

### 5.4. Further analysis

#### 5.4.1. The impact of internal control.

Internal control, as an internal governance mechanism, is essential for ensuring the quality of financial reporting and may interact with external auditor governance. Effective internal control may complement external auditing by enhancing its supervisory role, while poor internal control quality might fail to provide adequate governance, thereby requiring auditors to increase audit procedures to control audit risk, creating a substitute relationship [37,38].

To further verify the moderating effect of internal control quality on the relationship between client-auditor mismatch and earnings management using classification shifting, this study uses internal control index data from the Chinese DIB database, with the natural logarithm of the index value serving as a proxy variable for internal control level (*Inc*). A higher index value indicates better internal control quality. This index is constructed based on the five key elements explicitly stipulated in the *Basic Standards for Enterprise Internal Control*: control environment, risk assessment, control activities, information and communication, and monitoring. Each element is scored according to the firm’s degree of implementation, and the scores are subsequently adjusted to generate a comprehensive internal control index. A higher value of the internal control index indicates a more effective and well-developed internal control system in listed firms.

The internal control variable was included in the regression model, and the analysis was rerun. As shown in Table 7, the interaction term between upward mismatch and the internal control index has a coefficient of 0.049, which is significantly positively correlated at the 1% level. This suggests that better internal control quality and an upward mismatch are in a substitute relationship. This may be because clients in upward mismatch situations tend to be larger companies with more robust internal control mechanisms. In such cases, external auditors can fully leverage the internal control results of the company, reducing the need for extensive audit procedures, and leading to a substitute relationship.

**Table 7:** Internal Control, Client-Auditor Mismatch, and Earnings Management Using Classification Shifting.

	(1)	(2)
	*UE_CE*	*UE_CE*
*Misup*	−0.339***	
	(−3.720)	
*Misup×Inc*	0.049***	
	(3.511)	
*Misdown*		0.208***
		(3.070)
*Misdown×Inc*		−0.031***
		(−2.946)
*Inc*	0.119***	0.137***
	(19.076)	(20.294)
*Size*	0.016***	0.017***
	(18.362)	(19.268)
*Lev*	−0.269***	−0.271***
	(−52.206)	(−52.483)
*Dual*	0.009***	0.009***
	(5.064)	(4.975)
*Mborate*	0.001***	0.001***
	(15.121)	(15.115)
*Mb*	0.003***	0.003***
	(10.080)	(10.015)
*Firstshare*	−0.000***	−0.000***
	(−3.217)	(−3.268)
*Shrcr*	0.001***	0.001***
	(16.148)	(16.211)
*Boardsize*	0.008*	0.007
	(1.687)	(1.525)
*Indpend*	−0.000	−0.000
	(−1.318)	(−1.523)
*Opinion*	−0.106***	−0.106***
	(−23.289)	(−23.288)
*Constant*	−1.112***	−1.240***
	(−26.869)	(−28.136)
*Year Fixed Effects*	*Yes*	*Yes*
*Industry Fixed Effects*	*Yes*	*Yes*
*N*	28204	28204
*Adj.R2*	0.276	0.274

***, **, * Indicate significance levels at 0.01, 0.05, and 0.10, respectively. See Table 2 for variable definitions.

Conversely, the interaction term between downward mismatch and the internal control index has a coefficient of −0.031, significantly negatively correlated at the 1% level. This indicates that when internal control quality is poor, a downward mismatch poses a higher audit risk. As a result, auditors may implement more audit procedures to mitigate earnings management using classification shifting.

The moderating effect of internal control quality has produced mixed evidence in prior research. Ashbaugh-Skaife et al. [39] find that in firms with high-quality internal controls, auditors issue more accurate modified audit opinions. Effective internal controls reduce the information risk faced by auditors, enabling them to more precisely detect financial misstatements. In contrast, Bedard et al. [40] find that for firms with significant internal control deficiencies, auditors perceive higher risks and increase their audit effort by implementing additional non-standard audit procedures. This increased audit input improves auditors’ ability to detect covert earnings management practices, such as classification shifting and income manipulation. These findings are consistent with the results of this study.

#### 5.4.2. The influence of legal environment.

The legal environment in which a firm operates exerts a differentiated influence on both accrual-based and classification shifting earnings management. On one hand, it indirectly affects managerial incentives by altering the regulatory risks associated with accrual items; on the other hand, it directly shapes the legal costs inherent in classification shifting behavior. To capture regional legal enforcement intensity, this study adopts the “Development of Market Intermediaries and Legal Institutional Environment” sub-index from the *Marketization Index Report by Province in China*—a comprehensive annual series produced by the National Economic Research Institute (NERI) and published by Economic Science Press [41]. The Marketization Index provides province-level scores for various dimensions of market development, including institutional environment indicators relevant to legal enforcement, based on officially published annual reports. The sample is then divided into high and low legal environment groups according to the annual median score of this sub-index (High = 1; otherwise = 0).

Duong [42] provide empirical evidence that a sound legal system not only directly suppresses classification shifting but also moderates the negative relationship between audit quality and classification shifting. In regions with stronger legal enforcement, financial misstatements by clients are more likely to be detected by regulators, and auditors are more likely to be held legally accountable for negligence. Based on these insights, this study hypothesizes that in regions with a well-developed legal environment, upward auditor-client mismatches—where auditor expertise exceeds client sophistication—are more effective in curbing classification shifting behavior. In contrast, in regions with weaker legal enforcement, downward mismatches—where auditor expertise falls short of client complexity—may actually exacerbate classification shifting.

From the results in Table 8, the coefficient of *Misup* in Column (2) is –0.017 and statistically significant at the 1% level, indicating that, in regions with a stronger legal environment, upward mismatching reduces classification shifting earnings management. In Column (3), the coefficient of *Misdown* is 0.010 and significant at the 1% level, suggesting that, in regions with a weaker legal environment, downward mismatching increases classification shifting earnings management, which is consistent with the expected results. In mature capital markets, such as those of the United States and the United Kingdom, auditors face significantly higher litigation risks and reputational costs [43]. Classification shifting, as a quasi-misstatement, is particularly likely to trigger class-action lawsuits and regulatory sanctions, leaving little tolerance for such practices among auditors. Therefore, we conjecture that a similar pattern would likely hold in the United States, where the legal environment is relatively well developed.

**Table 8:** Client-Auditor Mismatch, Legal Environment, and Classification Shifting Earnings Management.

	(1)	(2)	(3)	(4)
	Weak Legal Environmen	Strong Legal Environment	Weak Legal Environmen	Strong Legal Environment
*Misup*	−0.008	−0.017***		
	(−1.549)	(−5.026)		
*Misdown*			0.010***	0.003
			(3.983)	(1.276)
*Size*	0.018***	0.021***	0.020***	0.018***
	(4.272)	(6.036)	(17.765)	(17.644)
*Lev*	−0.279***	−0.270***	−0.274***	−0.282***
	(−12.556)	(−13.157)	(−42.109)	(−46.169)
*Dual*	0.012***	0.008**	0.008***	0.011***
	(5.629)	(2.149)	(2.824)	(5.075)
*Mborate*	0.001***	0.001***	0.001***	0.001***
	(5.080)	(5.213)	(12.568)	(11.366)
*Mb*	−0.000*	0.000	0.000	−0.000
	(−1.934)	(0.204)	(0.224)	(−0.902)
*Firstshare*	−0.000	−0.000	−0.000**	0.000
	(−0.124)	(−0.921)	(−2.482)	(0.032)
*Shrcr*	0.001***	0.002***	0.002***	0.001***
	(5.369)	(6.268)	(14.825)	(11.434)
*Boardsize*	0.013	0.015	0.015**	0.012*
	(0.944)	(1.169)	(2.214)	(1.840)
*Indpend*	−0.000	−0.000	−0.000	−0.000
	(−0.093)	(−0.184)	(−0.116)	(−0.126)
*Opinion*	−0.142***	−0.160***	−0.163***	−0.142***
	(−8.990)	(−11.726)	(−29.317)	(−24.058)
*Constant*	−0.325***	−0.430***	−0.440***	−0.425***
	(−3.098)	(−4.644)	(−15.407)	(−14.709)
*Year Fixed Effects*	Yes	Yes	Yes	Yes
*Industry Fixed Effects*	Yes	Yes	Yes	Yes
*N*	15953	16439	16451	15965
*Adj.R2*	0.307	0.312	0.288	0.288

***, **, * Indicate significance levels at 0.01, 0.05, and 0.10, respectively. See Table 2 for variable definitions.

#### 5.4.3. The mediating effect of auditor industry expertise.

Based on the previous analysis, it is evident that there are differences in industry expertise between large and small auditors. The mismatch between clients and auditors may result in clients being paired with auditors who have varying levels of industry expertise. Therefore, we hypothesize that the client-auditor relationship might influence earnings management using classification shifting through the path of industry expertise. This study references the mediation effect model by Baron and Kenny [44] to further examine the mediating effect of auditor industry expertise (*Audprf*) in the relationship between client-auditor mismatch and earnings management using classification shifting.


$$Audprf_{i,t}=\partial_{\text{0}}+\partial_{\text{1}}Misup(Misdown{)}_{i,t}+\Sigma \partial_{j}Control_{j,i,t}+\Sigma Ind_{i,t}+\Sigma Year_{i,t}+\eta_{i,t}$$ (4)

$$UE\_CE_{i,t}=\partial_{\text{0}}+\partial_{\text{1}}Misup(Misdown{)}_{i,t}+\partial_{\text{2}}Audprf_{i,t}+\Sigma \partial_{j}Control_{j,i,t}+\Sigma Ind_{i,t}+\Sigma Year_{i,t}+\eta_{i,t}$$ (5)

The results of the mediation analysis are presented in Table 9. In column (1), the regression coefficient of upward mismatch (*Misup*) on auditor industry expertise (*Audprf*) is 0.026, which is significantly positive at the 1% level, indicating that an upward mismatch relationship enhances auditor industry expertise. After including the mediator variable, the regression coefficient of upward mismatch (*Misup*) on earnings management using classification shifting (*UE_CE*) is −0.018, which is also significant at the 1% level, suggesting that auditor industry expertise plays a partial mediating role.

**Table 9:** Mediating Effect of Auditor Industry Expertise.

	(1)	(2)	(3)	(4)
	*Audprf*	*UE_CE*	*Audprf*	*UE_CE*
*Misup*	0.026***	−0.018***		
	(30.802)	(−8.377)		
*Misdown*			−0.038***	0.010***
			(−56.859)	(5.511)
*Audprf*		0.076***		0.081***
		(5.501)		(5.654)
*Size*	0.012***	0.019***	0.012***	0.020***
	(37.726)	(23.633)	(39.674)	(24.856)
*Lev*	−0.012***	−0.294***	−0.013***	−0.296***
	(−6.301)	(−59.986)	(−7.175)	(−60.471)
*Dual*	0.002**	0.010***	0.001	0.010***
	(2.391)	(5.602)	(1.448)	(5.559)
*Mborate*	0.000***	0.001***	0.000***	0.001***
	(7.500)	(17.902)	(6.911)	(17.872)
*Mb*	0.000***	0.002***	0.000**	0.002***
	(3.334)	(9.158)	(2.127)	(9.242)
*Firstshare*	0.000	−0.000**	0.000**	−0.000**
	(1.275)	(−2.306)	(2.425)	(−2.295)
*Shrcr*	0.000***	0.001***	0.000***	0.001***
	(8.916)	(18.892)	(7.314)	(18.917)
*Boardsize*	0.005***	0.013***	0.006***	0.013***
	(2.580)	(2.820)	(3.415)	(2.727)
*Indpend*	0.000***	−0.000	0.000***	−0.000
	(2.640)	(−0.550)	(2.592)	(−0.606)
*Opinion*	−0.004**	−0.154***	−0.005***	−0.154***
	(−2.464)	(−38.476)	(−3.048)	(−38.433)
*Constant*	−0.228***	−0.422***	−0.227***	−0.442***
	(−28.500)	(−20.485)	(−29.728)	(−21.710)
*Year Fixed Effects*	Yes	Yes	Yes	Yes
*Industry Fixed Effects*	Yes	Yes	Yes	Yes
*N*	33958	32388	33958	32388
*Adj.R2*	0.165	0.291	0.216	0.290

***, **, * Indicate significance levels at 0.01, 0.05, and 0.10, respectively. See Table 2 for variable definitions.

In columns (3) and (4), the regression coefficient of downward mismatch (*Misdown*) on auditor industry expertise (*Audprf*) is −0.038, significantly negative at the 1% level, indicating that a downward mismatch relationship reduces auditor industry expertise. After including the mediator variable, the regression coefficient of downward mismatch (*Misdown*) on earnings management using classification shifting (*UE_CE*) is 0.010, significantly positive at the 1% level, again indicating a partial mediating effect. The mediating effect of auditor industry expertise is confirmed, demonstrating that the client-auditor mismatch relationship partially influences earnings management using classification shifting through the path of industry expertise.

## 6. Robustness tests

### 6.1. Excluding special samples from Ruihua CPA firm

Ruihua CPA Firm is one of the large domestic firms that has recently been frequently exposed in scandals. Following these scandals, there has been considerable public skepticism regarding Ruihua CPA Firm. To avoid the potential influence of this special sample on the overall results, we excluded Ruihua CPA Firm from the sample and re-conducted the analysis. Table 10 shows the regression results after excluding Ruihua CPA Firm. The results remain consistent with the previous conclusions, thus validating the hypotheses presented in this study.

**Table 10:** Regression Results After Removing Special Sample of Ruihua Certified Public Accountants.

	(1)	(2)
	*UE_CE*	*UE_CE*
*Misup*	−0.017***	
	(−7.476)	
*Misdown*		0.005***
		(2.826)
*Size*	0.021***	0.022***
	(25.061)	(26.769)
*Lev*	−0.298***	−0.301***
	(−59.052)	(−59.694)
*Dual*	0.010***	0.009***
	(5.336)	(5.225)
*Mborate*	0.001***	0.001***
	(17.845)	(17.754)
*Mb*	0.003***	0.003***
	(10.664)	(10.654)
*Firstshare*	−0.000*	−0.000*
	(−1.742)	(−1.664)
*Shrcr*	0.001***	0.001***
	(18.892)	(18.825)
*Boardsize*	0.014***	0.014***
	(2.983)	(2.930)
*Indpend*	−0.000	−0.000
	(−0.670)	(−0.721)
*Opinion*	−0.156***	−0.156***
	(−37.671)	(−37.693)
*Constant*	−0.447***	−0.473***
	(−21.281)	(−22.851)
*Year Fixed Effects*	Yes	Yes
*Industry Fixed Effects*	Yes	Yes
*N*	28204	28204
*Adj.R2*	0.276	0.274

***, **, * Indicate significance levels at 0.01, 0.05, and 0.10, respectively. See Table 2 for variable definitions.

### 6.2. Incorporating auditor tenure and big 4 auditors

We further incorporate auditor tenure and the international Big Four auditor indicator into the regression models and re-estimate the analyses. The results remain robust, as reported in Table 11. We also review classification-shifting earnings management models and find no substantive differences from the model adopted in this study. Accordingly, we conclude that minor model variations do not materially affect our results. To eliminate the influence of government subsidies, we excluded non-operating income when calculating the total revenue–related variables (ΔSales and Neg_ΔSales) in Model (2) and re-estimated the regressions. These results are consistent with the main findings but are not reported in the paper.

**Table 11:** Incorporating Auditor Tenure and Big 4 Auditors.

	(1)	(2)	(3)	(4)
	Incorporating Auditor Tenure		Incorporating Big 4 Auditors	
*Misup*	−0.017***		−0.015***	
	(−7.7165)		(−6.8718)	
*Misdown*		0.007***		0.006***
		(3.9902)		(3.3652)
*Turn*	0.001***	0.001***		
	(4.0691)	(4.1734)		
*Big 4*			−0.002	−0.001
			(−0.7206)	(−0.2575)
*Size*	0.020***	0.021***	0.020***	0.021***
	(24.4880)	(25.9240)	(24.4240)	(25.4320)
*Lev*	−0.294***	−0.297***	−0.302***	−0.304***
	(−58.6277)	(−59.1723)	(−61.4729)	(−61.9116)
*Dual*	0.010***	0.010***	0.010***	0.010***
	(5.3954)	(5.3301)	(5.8605)	(5.8076)
*Mborate*	0.001***	0.001***	0.001***	0.001***
	(18.1052)	(18.0739)	(17.5340)	(17.4967)
*Mb*	0.002***	0.002***	0.003***	0.003***
	(8.7631)	(8.8248)	(11.6453)	(11.6430)
*Firstshare*	−0.000**	−0.000**	−0.000**	−0.000**
	(−2.2041)	(−2.1393)	(−2.4773)	(−2.4098)
*Shrcr*	0.001***	0.001***	0.001***	0.001***
	(19.1279)	(19.1033)	(18.4496)	(18.3905)
*Boardsize*	0.014***	0.013***	0.012**	0.011**
	(2.8262)	(2.7614)	(2.4819)	(2.4144)
*Indpend*	−0.000	−0.000	−0.000	−0.000
	(−0.3366)	(−0.3902)	(−0.3661)	(−0.4528)
*Opinion*	−0.158***	−0.159***	−0.128***	−0.128***
	(−38.4089)	(−38.4078)	(−29.8649)	(−29.8953)
*Constant*	−0.433***	−0.456***	−0.439***	−0.457***
	(−20.5585)	(−21.9329)	(−20.9611)	(−21.9635)
*Year Fixed Effects*	Yes	Yes	Yes	Yes
*Industry Fixed Effects*	Yes	Yes	Yes	Yes
N	31711	31711	31467	31467
Adj.R2	0.286	0.285	0.281	0.280

***, **, * Indicate significance levels at 0.01, 0.05, and 0.10, respectively. See Table 2 for variable definitions.

### 6.3. Replacing the dependent variable

In this study, following McVay [3], we replaced the measure of unexpected core earnings level (*UE_CE*) with the change in unexpected core earnings level (*UE_*Δ*CE*) as a new measure of classification shift earnings management, and performed a robustness check. The measurement model for the dependent variable was revised to Model (6), as follows:


$$\begin{array}{c} \Delta Ce_{t}=\partial_{\text{0}}+\partial_{\text{1}}Ce_{t-1}+\partial_{\text{2}}\Delta Ce_{t-1}+\partial_{\text{3}}\Delta Ato_{t}+\partial_{\text{4}}Accruals_{t}+\partial \text{5}Accruals_{t-1}\\ +\partial_{\text{6}}\Delta Sales_{t}+\partial_{\text{7}}Neg\_\Delta Sales_{t}+\varepsilon_{t} \end{array}$$ (6)

We performed regression analysis using Model (6) to obtain residuals that measure the change in unexpected core earnings level (*UE_*Δ*CE*). We then substituted this new dependent variable into Model (3) for regression analysis. The results, as shown in Table 12, are consistent with the previous conclusions, confirming the validity of the hypotheses in this study.

**Table 12:** Client-Auditor Mismatch and Earnings Management Using Classification Shifting.

	(1)	(2)
	*UE_ΔCE*	*UE_ΔCE*
*Misup*	−0.023***	
	(−9.151)	
*Misdown*		0.009***
		(4.789)
*Size*	0.021***	0.022***
	(22.821)	(24.532)
*Lev*	−0.270***	−0.273***
	(−49.427)	(−50.079)
*Dual*	0.008***	0.008***
	(4.087)	(3.976)
*Mborate*	0.001***	0.001***
	(15.276)	(15.289)
*Mb*	0.002***	0.002***
	(8.031)	(8.081)
*Firstshare*	−0.000	−0.000
	(−1.253)	(−1.191)
*Shrcr*	0.001***	0.001***
	(16.050)	(16.047)
*Boardsize*	0.011**	0.011**
	(2.189)	(2.072)
*Indpend*	−0.000	−0.000
	(−0.975)	(−1.071)
*Opinion*	−0.126***	−0.126***
	(−28.887)	(−28.912)
*Constant*	−0.434***	−0.465***
	(−19.044)	(−20.651)
*Year Fixed Effects*	Yes	Yes
*Industry Fixed Effects*	Yes	Yes
*N*	28569	28569
*Adj.R2*	0.231	0.229

***, **, * Indicate significance levels at 0.01, 0.05, and 0.10, respectively. See Table 2 for variable definitions.

### 6.4. Robustness to alternative cutoff probability thresholds

To examine the robustness of our findings with respect to the choice of the cutoff probability threshold, we reclassify client-auditor mismatch using three alternative thresholds: 0.464, 0.484 (the baseline threshold), and 0.504, and repeat the main regression analyses accordingly. The results, reported in Table 13, show that under all alternative threshold settings, the estimated coefficients on client-auditor mismatch remain consistent in sign and are statistically significant at the 1% level. Moreover, the magnitude of the coefficients exhibits no economically meaningful differences across thresholds. These findings indicate that our main conclusions are stable and not sensitive to small variations in the cutoff probability threshold.

**Table 13:** Sensitivity Analysis of Critical Probability.

	Baseline threshold	Other threshold	Other threshold
	0.484	0.464	0.504
*Mismatch_0484*	0.049^***^		
	(0.004)		
*Mismatch_0504*		0.048^***^	
		(0.004)	
*Mismatch_0464*			0.058^***^
			(0.004)
*Dual*	0.009^***^	0.008^***^	0.008^***^
	(0.003)	(0.003)	(0.003)
*Mborate*	0.001^***^	0.001^***^	0.001^***^
	(0.000)	(0.000)	(0.000)
*Mb*	0.002^***^	0.002^***^	0.003^***^
	(0.001)	(0.001)	(0.001)
*Firstshare*	−0.000	−0.000	−0.000
	(0.000)	(0.000)	(0.000)
*Shrcr*	0.001^***^	0.001^***^	0.001^***^
	(0.000)	(0.000)	(0.000)
*Boardsize*	0.008	0.009	0.008
	(0.009)	(0.009)	(0.009)
*Indpend*	−0.000	−0.000	−0.000
	(0.000)	(0.000)	(0.000)
*Opinion*	−0.114^***^	−0.115^***^	−0.111^***^
	(0.012)	(0.012)	(0.012)
*Constant*	−0.307^***^	−0.292^***^	−0.313^***^
	(0.048)	(0.049)	(0.047)
*Year Fixed Effects*	Yes	Yes	Yes
*Industry Fixed Effects*	Yes	Yes	Yes
*N*	28568	28568	28568
*Adj.R2*	*0.246*	*0.244*	*0.247*

***, **, * Indicate significance levels at 0.01, 0.05, and 0.10, respectively. See Table 2 for variable definitions.

In addition, we conduct a chi-square test to examine the discriminatory power of the threshold-based classification. The results, reported in Table 14, show that the p-value equals 0.000 (< 0.001), indicating that the threshold-based indicator (Mismatch_0484) and the baseline mismatch label (MISMATCH) are highly significantly dependent. This indicates that the classification based on the 0.484 threshold is systematically related to the benchmark mismatch measure rather than being random, supporting the statistical validity of the chosen cutoff.

**Table 14:** Chi-square Test.

Mismatch_0484	MISMATCH		Total
	0	1	
0	8,544	4,224	12,768
1	16,179	21,480	37,659
Total	24,723	25,704	50,427

In terms of classification accuracy, the correct classification rate—defined as the number of observations for which the threshold-based classification is consistent with the baseline label divided by the total sample size—is 30,024/ 50,427, corresponding to approximately 59.54%. This level of accuracy falls within an acceptable range and indicates that the 0.484 threshold can effectively identify about 60% of the observations, demonstrating a reasonable level of classification capability.

### 6.5. Endogeneity testing

#### 6.5.1. Propensity score matching method.

To address potential endogeneity issues, we paired samples of mismatched and matched clients and auditors using the propensity score matching (PSM) method. After completing the matching, we re-conducted the regression analysis with the matched samples. The results, presented in Table 15, are consistent with the previous findings, confirming that the regression results, after addressing endogeneity, support the earlier conclusions and demonstrate the robustness of our results.

**Table 15:** Client-Auditor Mismatch and Earnings Management Using Classification Shifting (PSM).

	(1)	(2)
	*UE_CE*	*UE_CE*
*Misup*	−0.016**	
	(−4.532)	
*Misdown*		0.007***
		(2.880)
*Size*	0.018***	0.023***
	(8.332)	(17.713)
*Lev*	−0.221***	−0.330***
	(−21.431)	(−41.313)
*Dual*	0.005	0.007***
	(1.421)	(2.681)
*Mborate*	0.001***	0.001***
	(9.052)	(12.301)
*Mb*	−0.000	0.006***
	(−0.845)	(12.572)
*Firstshare*	−0.000	−0.000*
	(−0.222)	(−1.967)
*Shrcr*	0.002***	0.001***
	(10.876)	(11.783)
*Boardsize*	0.004	0.016**
	(0.362)	(2.284)
*Indpend*	−0.000	0.000
	(−1.065)	(0.124)
*Opinion*	−0.174**	−0.148***
	(−22.812)	(−20.740)
*Constant*	−0.394***	−0.499***
	(−7.656)	(−15.052)
*Year Fixed Effects*	Yes	Yes
*Industry Fixed Effects*	Yes	Yes
*N*	8386	12909
*Adj.R2*	0.274	0.260

***, **, * Indicate significance levels at 0.01, 0.05, and 0.10, respectively. See Table 2 for variable definitions.

#### 6.5.2. Exogenous shock.

This study selects the 2020 New Securities Law as an exogenous shock to mitigate endogeneity concerns. The new law strengthens the legal responsibilities of auditing firms and increases auditors’ professional risk. It also tightens penalties for corporate misconduct, raising the maximum fines for financial fraud and audit malpractice from RMB 600,000 to RMB 20 million, thereby pressuring auditors to enhance audit quality and exercise greater caution in client selection.

Firms with matched and mismatched auditors are treated as the control and treatment groups, respectively. The policy was officially implemented in 2020, allowing a natural experiment around that year. The variable Treat indicates whether a client-auditor mismatch exists (Treat = 1 if mismatched; 0 otherwise).

If client-auditor mismatches indeed induce classification-shifting earnings management, this phenomenon should significantly decline after the implementation of the New Securities Law. The new policy increases audit risk, prompting some firms to reappoint auditors, which generates an exogenous variation in the mismatch variable. The re-estimated results are presented in Table 16. The results show that this effect is significantly weakened after regulatory strengthening, providing evidence that mismatches indeed lead to classification-shifting behavior.

**Table 16:** Difference-in-Differences Regression Results.

	(1)
	UE_CE
*Post*	0.007
	(1.44)
*Treat*	0.001
	(0.14)
*Treat×Post*	−0.016***
	(−4.8363)
*Size*	0.021***
	(26.9285)
*Lev*	−0.298***
	(−60.3876)
*Dual*	0.009***
	(5.3680)
*Mborate*	0.001***
	(17.7902)
*Mb*	0.002***
	(8.8083)
*Firstshare*	−0.000*
	(−1.9532)
*Shrcr*	0.001***
	(18.5854)
*Boardsize*	0.014***
	(2.8644)
*Indpend*	−0.000
	(−0.5565)
*Opinion*	−0.159***
	(−39.1916)
*Constant*	−0.456***
	(−22.3802)
*Year Fixed Effects*	YES
*Industry Fixed Effects*	YES
N	32416
Adj.R2	0.288

***, **, * Indicate significance levels at 0.01, 0.05, and 0.10, respectively. See Table 2 for variable definitions.

### 6.6. Fixed effects model

We more thoroughly employ firm fixed effects to more effectively control for unobserved time-invariant firm characteristics, thereby enabling a more reliable identification of the causal impact of client–auditor mismatches on earnings management. The regression results are presented in Table 17. Both upward mismatches (*Misup*) and downward mismatches (*Misdown*) are significant at the 1% level, largely consistent with the baseline regression results, suggesting that the main findings of the study remain robust after controlling for firm-level time-invariant characteristics and are not significantly affected by omitted variable bias.

**Table 17:** Fixed Effects Model.

	(1)	(2)
	UE_CE	UE_CE
*Misup*	−0.022***	
	(−9.999)	
*Misdown*		0.013***
		(6.411)
*Size*	0.014***	0.015***
	(10.172)	(10.796)
*Lev*	−0.224***	−0.227***
	(−35.341)	(−35.693)
*Dual*	0.004**	0.004**
	(2.157)	(2.055)
*Mborate*	0.001***	0.001***
	(7.013)	(6.863)
*Mb*	0.001**	0.001**
	(2.328)	(2.384)
*Firstshare*	0.000	0.000
	(0.324)	(0.382)
*Shrcr*	0.001***	0.001***
	(11.032)	(10.624)
*Boardsize*	0.008	0.007
	(1.134)	(0.969)
*Indpend*	0.000	0.000
	(1.068)	(1.061)
*Opinion*	−0.122***	−0.122***
	(−33.093)	(−33.190)
*Constant*	−0.274***	−0.294***
	(−7.833)	(−8.416)
*Year Fixed Effects*	Yes	Yes
*Industry Fixed Effects*	Yes	Yes
*Firm Fixed Effects*	Yes	Yes
*N*	31931	31931
Adj.R2	0.590	0.589

***, **, * Indicate significance levels at 0.01, 0.05, and 0.10, respectively. See Table 2 for variable definitions.

### 6.7. Clustered regressions

To mitigate potential heteroskedasticity, serial correlation, and other issues at the firm level that may affect statistical inference, this study adjusts the standard errors of all regression estimates for clustering at the firm level. As reported in Table 18, after clustering adjustment, the signs and significance levels of the coefficients for the core explanatory variables remain largely unchanged, indicating that the baseline regression results are robust.

**Table 18:** Clustered Regressions.

	(1)	(2)
	UE_CE	UE_CE
*Misup*	−0.017***	
	(−4.2139)	
*Misdown*		0.007**
		(2.3661)
*Size*	0.020***	0.021***
	(11.7068)	(12.3618)
*Lev*	−0.295***	−0.297***
	(−23.5555)	(−23.6592)
*Dual*	0.010***	0.010***
	(3.3746)	(3.3335)
*Mborate*	0.001***	0.001***
	(10.4695)	(10.4434)
*Mb*	0.002***	0.002***
	(3.1997)	(3.2210)
*Firstshare*	−0.000	−0.000
	(−1.0238)	(−1.0018)
*Shrcr*	0.001***	0.001***
	(9.7541)	(9.7374)
*Boardsize*	0.014	0.013
	(1.5460)	(1.5125)
*Indpend*	−0.000	−0.000
	(−0.2634)	(−0.2920)
*Opinion*	−0.159***	−0.159***
	(−12.9180)	(−12.9761)
*Constant*	−0.400***	−0.421***
	(−9.3178)	(−10.0377)
*Year Fixed Effects*	YES	YES
*Industry Fixed Effects*	YES	YES
*N*	32416	32416
Adj.R2	0.288	0.288

***, **, * Indicate significance levels at 0.01, 0.05, and 0.10, respectively. See Table 2 for variable definitions. Standard errors are clustered at the firm level to account for within-firm serial correlation.

## 7. Conclusions and implications

This study provides new evidence on the role of client–auditor mismatches in shaping earnings management through classification shifting. Specifically, an upward mismatch significantly constrains classification-shifting behavior, whereas a downward mismatch facilitates such behavior. Further analysis shows that high-quality internal control partially substitutes for auditor oversight under upward mismatches, likely because stronger internal control systems improve information reliability and reduce auditors’ reliance on extensive substantive procedures. In addition, auditor industry expertise partially mediates the relationship between client–auditor mismatches and classification-shifting earnings management, highlighting the importance of auditors’ domain-specific knowledge in detecting subtle reporting manipulation.

The findings offer important economic and organizational insights. Although classification shifting does not affect reported net income, it alters the composition of earnings and undermines the transparency and interpretability of financial statements. In the Chinese context, where managerial compensation and promotion incentives are closely linked to measures of “core earnings,” firms face dual pressures from performance targets and regulatory constraints—including ST delisting rules, refinancing thresholds, and penalties for financial misreporting. Under downward mismatches, these pressures may be further intensified because auditors with inadequate expertise or resources may struggle to identify complex transactions. As a result, classification shifting becomes a relatively low-cost and low-detection-risk strategy for achieving performance benchmarks. These findings highlight the interaction among external regulation, audit market structure, and corporate governance mechanisms, illustrating how institutional environments shape managerial reporting decisions and earnings management behavior.

From a practical and policy perspective, several implications emerge. Firms should consider their own characteristics—such as operational complexity, organizational scale, and risk exposure—when selecting auditors, rather than relying solely on audit fees or brand reputation. Audit committees should actively evaluate the alignment between auditors’ capabilities and the firm’s audit demands, maintain regular communication with external auditors, and report potential mismatches to the board when audit quality may be at risk. Regulatory bodies, such as the China Securities Regulatory Commission (CSRC), could further enhance market transparency by encouraging or requiring disclosures related to client–auditor matching conditions, including auditor industry expertise, client operational complexity, and the allocation of audit resources. Firms exhibiting pronounced mismatches—such as large, complex clients audited by small firms—may warrant differentiated regulatory scrutiny, including enhanced risk assessments and more frequent inquiries, to deter opportunistic reporting practices.

This study also contributes to theory by identifying the mechanisms through which mismatches in client–auditor supply and demand influence classification-shifting behavior. It highlights the roles of internal controls and auditor expertise as both mediating and substituting factors, offering a more nuanced understanding of audit quality and corporate governance in China. Importantly, the findings are context-dependent: although they shed light on mechanisms within China’s regulatory and institutional framework, they should not be generalized to other emerging markets without further comparative evidence. Future research could extend this framework to cross-country settings, examine the external validity of the effects documented here, and explore additional dimensions of audit quality—such as partner-level expertise or the intensity of audit effort.

Finally, several limitations merit consideration. The analysis relies on data from Chinese A-share firms, which may contain measurement errors or incomplete disclosures. The study period (2008–2022) spans several regulatory reforms that could influence earnings-management practices. Although year fixed effects were included, some policy changes may not be fully captured. These limitations provide avenues for future research to deepen our understanding of the interplay among audit quality, corporate governance, and earnings management.

## Supporting information

S1 file Data. (DTA)

S2 file Internal control index. (DTA)

## References

[1] Bushman RM, Smith AJ. Financial accounting information and corporate governance. J Account Econ. 2001;32(1–3):237–333.

[2] Haw IM, Ho SS, Li AY. Corporate governance and earnings management by classification shifting. Contemp Account Res. 2011;28(2):517–53. doi: 10.1111/j.1911-3846.2010.01059.x

[3] McVay SE. Earnings management using classification shifting: An examination of core earnings and special items. Account Rev. 2006;81(3):501–31. doi: 10.2308/accr.2006.81.3.501

[4] Sloan RG. Do stock prices fully reflect information in accruals and cash flows about future earnings? Account Rev. 1996;71(2):289–315.

[5] Dechow P, Ge W, Schrand C. Understanding earnings quality: A review of the proxies, their determinants and their consequences. J Account Econ. 2010;50(2–3):344–401. doi: 10.1016/j.jacceco.2010.09.001

[6] Hribar P, Nichols DC. The use of unsigned earnings quality measures in tests of earnings management. J Account Res. 2007;45(5):1017–53. doi: 10.1111/j.1475-679X.2007.00259.x

[7] Rawashdeh M, AlKhalailah M, Zaidan H. The impact of earnings management on the value relevance of earnings: Empirical evidence from Jordan. Studies in Systems, Decision and Control. Cham: Springer. 2024. p. 619–28.

[8] Burlacu G, Robu I-B, Munteanu I. Exploring the Influence of Earnings Management on the Value Relevance of Financial Statements: Evidence from the Bucharest Stock Exchange. IJFS. 2024;12(3):72. doi: 10.3390/ijfs12030072

[9] Garel A. Myopic market pricing and managerial myopia. Business Fin & Account. 2017;44(9–10):1194–213. doi: 10.1111/jbfa.12262

[10] Becker CL, DeFond ML, Jiambalvo J, Subramanyam KR. The effect of audit quality on earnings management. Contemp Account Res. 1998;15(1):1–24. doi: 10.1111/j.1911-3846.1998.tb00547.x

[11] Nelson MW, Elliott JA, Tarpley RL. Evidence from auditors about managers’ and auditors’ earnings management decisions. Account Rev. 2002;77(S1):175–202. doi: 10.2308/accr.2002.77.s-1.175

[12] Fan JPH, Wong TJ. Do external auditors perform a corporate governance role in emerging markets? Evidence from East Asia. J Account Res. 2005;43(1):35–72. doi: 10.1111/j.1475-679X.2004.00162.x

[13] Brown SV, Knechel WR. Auditor-client compatibility and audit firm selection. J Account Res. 2016;54(3):725–75. doi: 10.1111/1475-679X.12105

[14] Bills KL. The effects of significant changes in auditor clientele and auditor-client mismatches on audit quality [dissertation]. Norman (OK): University of Oklahoma; 2012. Available from: https://hdl.handle.net/11244/320310

[15] Velte P, Stiglbauer M. Impact of audit committees with independent financial experts on accounting quality. Probl Perspect Manag. 2011;9(4):17–33.

[16] Lai KW, Leung PW. Auditor-client match: timing of auditor change following mismatch and improvement through the change. Manag Audit J. 2023;38(5):579–601.

[17] Bruynseels L, Cardinaels E. The audit committee: Management watchdog or personal friend of the CEO? Account Rev. 2014;89(1):113–45. doi: 10.2308/accr-50601

[18] Guan Y, Su LN, Wu D, Yang Z. Do school ties between auditors and client executives influence audit outcomes? J Account Econ. 2016;61(2–3):506–25. doi: 10.1016/j.jacceco.2015.09.003

[19] Choi J-H, Kim J-B, Qiu AA, Zang Y. Geographic Proximity between Auditor and Client: How Does It Impact Audit Quality? Auditing. 2012;31(2):43–72. doi: 10.2308/ajpt-10241

[20] Francis JR, Golshan N, Hallman NJ. Does distance matter? An investigation of partners who audit distant clients and the effects on audit quality. Contemp Account Res. 2022;39(2):947–81. doi: 10.1111/1911-3846.12744

[21] Low KY. The effects of industry specialization on audit risk assessments and audit‐planning decisions. Account Rev. 2004;79(1):201–19. doi: 10.2308/accr.2004.79.1.201

[22] Hammersley JS. Pattern identification and industry‐specialist auditors. Account Rev. 2006;81(2):309–36. doi: 10.2308/accr.2006.81.2.309

[23] Hutton AP, Marcus AJ, Tehranian H. Opaque financial reports, R2, and crash risk. J Financ Econ. 2009;94(1):67–86. doi: 10.1016/j.jfineco.2008.10.003

[24] Liu M, Sun J. The impact of COVID‑19 pandemic on earnings management and the value relevance of earnings: US evidence. Manag Audit J. 2022;37(7):850–68. doi: 10.1108/MAJ-05-2021-3149

[25] Ha K, Thomas WB. Classification Shifting and Earnings Predictability. J Account Audit Finance. 2023;40(3):1033–60. doi: 10.1177/0148558x231210601

[26] Fan Y, Barua A, Cready WM, Thomas WB. Managing earnings using classification shifting: Evidence from quarterly special items. Account Rev. 2010;85(4):1303–23. doi: 10.2308/accr.2010.85.4.1303

[27] Rehman AU, Yaqub A, Ahsan T, Rao ZUR. Earnings management using classification shifting of revenues: evidence from Chinese-listed firms. J Account Emerg Econ. 2024;14(5):1061–83. doi: 10.1108/JAEE-04-2022-0118

[28] Joo JH, Chamberlain SL. The effects of governance on classification shifting and compensation shielding. Contemp Account Res. 2017;34(4):1779–811. doi: 10.1111/1911-3846.12331

[29] Chen H, Chen JZ, Lobo GJ, Wang Y. Effects of audit quality on earnings management and cost of equity capital: Evidence from China. Contemp Account Res. 2011;28(3):892–925. doi: 10.1111/j.1911-3846.2011.01088.x

[30] DeAngelo LE. Auditor size and audit quality. J Account Econ. 1981;3(3):183–99. doi: 10.1016/0165-4101(81)90002-1

[31] Reichelt KJ, Wang D. National and office-specific measures of auditor industry expertise and effects on audit quality. J Account Res. 2010;48(3):647–86.

[32] Chi W, Douthett EB Jr, Lisic LL. Client importance and audit partner independence. J Account Public Policy. 2012;31(3):320–36. doi: 10.1016/j.jaccpubpol.2011.08.009

[33] Trompeter G. The effect of partner compensation schemes and generally accepted accounting principles on audit partner judgment. Auditing. 1994;13(2):69–89.

[34] Carcello JV, Hermanson DR, Huss HF. Going-Concern Opinions: The Effects of Partner Compensation Plans and Client Size. Auditing: A J Pract The. 2000;19(1):67–77. doi: 10.2308/aud.2000.19.1.67

[35] Shu SZ. Auditor resignations: Clientele effects and legal liability. J Account Econ. 2000;29(2):173–205. doi: 10.1016/S0165-4101(00)00019-7

[36] Liu X, Yang J, Di R, Li M. CFO Tenure and Classification Shifting: Evidence from China. Emerg Mark Finance Trade. 2022;58(6):1578–89. doi: 10.1080/1540496x.2021.1904879

[37] Prawitt DF, Smith JL, Wood DA. Internal audit quality and earnings management. Account Rev. 2009;84(4):1255–80. doi: 10.2308/accr.2009.84.4.1255

[38] Chi G, Gooda AR. Internal control, debt risk, CEO education and earnings management evidence from China. J Financ Report Account. 2024;22(1):52–78. doi: 10.1108/JFRA-05-2023-0237

[39] Ashbaugh-Skaife H, Collins D, Kinney W. The effect of SOX internal control deficiencies and their remediation on accrual quality. Acct Rev. 2008;83(1):217–50. doi: 10.2308/accr.2008.83.1.217

[40] Bedard JC, Johnstone KM. Audit Partner Tenure and Audit Planning and Pricing. A J Pract Theory. 2010;29(2):45–70. doi: 10.2308/aud.2010.29.2.45

[41] Wang X, Fan G, Yu JW. Marketization Index Report by Provinces in China. Beijing, China: Social Science Literature Press; 2021.

[42] Duong Thi C. Audit Quality, Institutional Environments, and Earnings Management: An Empirical Analysis of New Listings. Sage Open. 2023;13(2). doi: 10.1177/21582440231180672

[43] DeFond ML, Francis JR, Hallman NJ. Awareness of SEC enforcement and auditor reporting decisions. Contemp Account Res. 2018;35(1):277–313. doi: 10.1111/1911-3846.12352

[44] Baron RM, Kenny DA. The moderator-mediator variable distinction in social psychological research: conceptual, strategic, and statistical considerations. J Pers Soc Psychol. 1986;51(6):1173–82. doi: 10.1037//0022-3514.51.6.1173 3806354

